# Death rate of patients admitted to a Brazilian ICU on weekends and holidays

**DOI:** 10.1186/cc13224

**Published:** 2014-03-17

**Authors:** P Batista, R Passos, R Oliveira, M Ribeiro, F Alves

**Affiliations:** 1 Hospital Sao Rafael, Salvador Bahia, Brazil

## Introduction

Several studies conducted in a number of different populations indicate that patients admitted to hospitals on weekends have a higher mortality rate. Many factors have been proposed to explain these observations and in most studies, however, this effect disappeared after controlling for illness severity. We undertook the present study to explore the effect that day of ICU admission may have on death rate.

## Methods

We analyzed 11,230 electronic records from patients admitted from 1 January 2006 to 30 June 2013. The ICU admission dates were categorized as normal weekdays and as weekends and holidays. The dependent variables were ICU mortality and hospital mortality. The chi- square test and Student *t *test were used as appropriate and significant association was accepted when *P *< 0.05. Multiple logistic regression (backward conditional) was used accepting a variable when *P *< 0.05 and rejecting a variable with *P *> 0.1. SPSS version 19.0 was used.

## Results

Forty-nine percent of these patients were female, 53% was admitted immediately after surgery, 56% had <2 days of previous hospital admission, and 19.2% were admitted during weekends and holidays. Mortality in the ICU for admissions on weekends and holidays was 24% and was 13% for those admitted on weekdays (OR = 2.23, 95% CI 2.02 to 2.47; *P *< 0.0001). The hospital mortality was respectively 35% and 20% (OR = 2.23, 95% CI 2.02 to 2.47; *P *< 0.0001). Differences between patients admitted on weekends and on weekdays were found in the group admitted after surgery (respectively 10% and 30%, *P *< 0.0001), originating from the emergency unit (respectively 30% and 16%; *P *< 0.0001), originating from step-down units (respectively 31% and 17%; *P *< 0.0001), patients from clinical teams compared with surgical teams (respectively 28% and 13%; *P *< 0.0001), previous hospital length of stay lower versus higher than 2 days (respectively 15% and 25%; *P *< 0.0001) and age ≥65 years (respectively 21% and 18%; *P *< 0.0001). The mortality ratios were significantly different between these groups. Multiple logistic regression showed that the inclusion of other variables reduces the odds ratio associated with admission on weekends and holidays to 1.22 (95% CI 1.08 to 1.39, *P *< 0.002) for ICU mortality and to 1.23 (95% CI 1.09 to 1.38, *P *< 0.001).

## Conclusion

The higher death rate on weekends and holidays may be related to case mix. The interplay of other variables possibly related either to admission on weekends or higher death rate should be sought.

